# Capparis spinosa improves non-alcoholic steatohepatitis through down-regulating SREBP-1c and a PPAR*α*-independent pathway in high-fat diet-fed rats

**DOI:** 10.1186/s13104-022-06205-x

**Published:** 2022-10-03

**Authors:** Rasoul Akbari, Hamid Yaghooti, Mohammad Taha Jalali, Laya Sadat Khorsandi, Narges Mohammadtaghvaei

**Affiliations:** 1 Hyperlipidemia Research Center, Ahvaz Jundishapur University of Medical Sciences, Ahvaz, Iran; 2 Department of Laboratory Sciences, School of Allied Medical Sciences, Ahvaz Jundishapur University of Medical Sciences, Ahvaz, Iran; 3 Cellular and Molecular Research Center, Ahvaz Jundishapur University of Medical Sciences, Ahvaz, Iran

**Keywords:** Capparis spinosa, NASH, SREBP-1c, ACC, PPARα, CPT1, Fenofibrate

## Abstract

**Objective:**

Non-alcoholic steatohepatitis (NASH) has become a global medical problem. Currently, there is no approved pharmacologic treatment for this condition. Previous studies have suggested that in the pathogenesis of this disease, regulatory pathways associated with de novo lipogenesis and β-oxidation pathways genes are misregulated. Capparis spinosa (CS) belongs to the family of Capparidaceae and is a traditional plant used to treat various diseases, particularly dyslipidemia. The compounds and extracts of this plant in In vivo and in vitro studies resulted in a reduction in lipid profiles and glucose. However, the mechanism of these effects remains unknown. This study aimed to evaluate the effects of (CS) fruit extract on NASH compared to fenofibrate and explored the related molecular mechanism.

**Results:**

In the rats (n = 40) model of NASH, biochemical and histopathological examinations showed that liver steatosis, inflammation, and hepatic fibrosis were markedly attenuated in response to CS and fenofibrate interventions. At the molecular level, CS treatment down-regulated sterol regulatory element-binding protein-1c (SREBP-1c) (p < 0.001), acetyl-CoA carboxylase (ACC) (p < 0.001), and up-regulated Carnitine palmitoyltransferase I (CPT1) expression (p < 0.001). In conclusion, CS has favorable therapeutic effects for NASH, which was associated with ameliorating steatosis and fibrosis via regulation of the DNL and β-oxidation pathway genes.

**Graphical Abstract:**

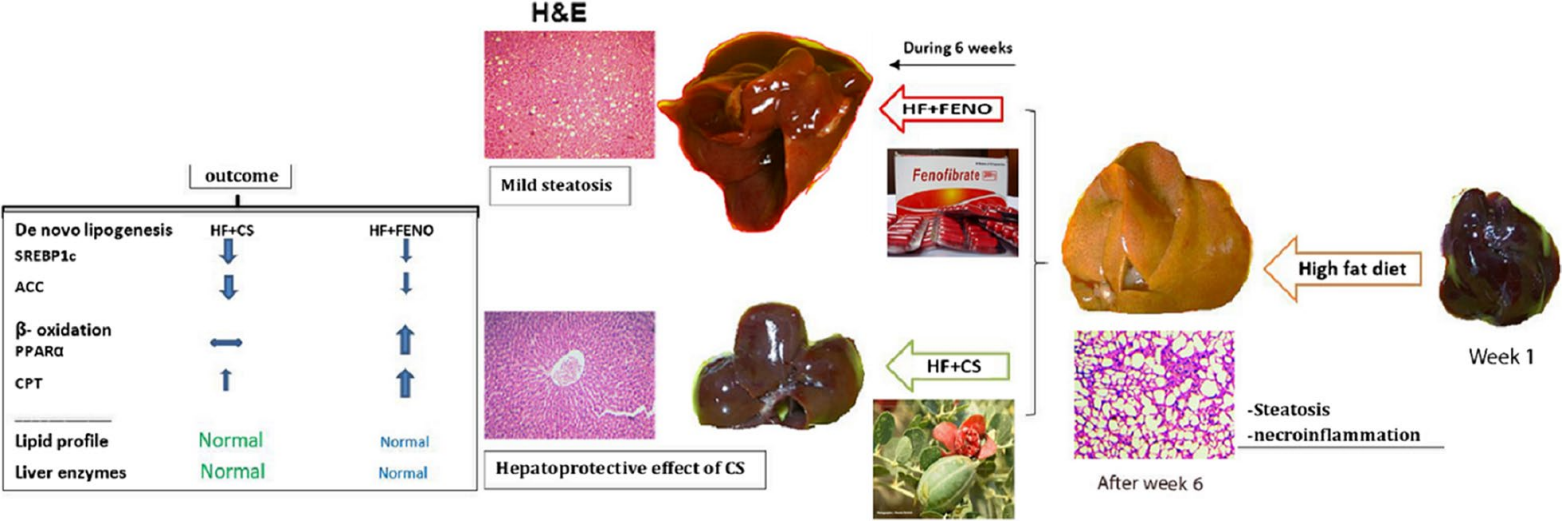

**Supplementary Information:**

The online version contains supplementary material available at 10.1186/s13104-022-06205-x.

## Introduction

Non-alcoholic fatty liver disease (NAFLD) is among the most common types of liver disease and is becoming an important public health problem all over the world [1]. Hepatic injury in NAFLD results in the release of plasma aminotransferases (AST and ALT]) into the circulation, and these are routinely determined as the classic markers of NAFLD. Nonalcoholic steatohepatitis (NASH) is a progressive type of NAFLD [2]. Disorders related to metabolic syndrome including obesity and insulin resistance lead to hepatic accumulation of triglycerides and free fatty acids that contribute to the development of liver inflammation and NASH [3]. Several key transcription factors including Sterol Regulatory Element Binding Proteins (SREBP) such as SREBP-1a, SREBP-1c, and SREBP-2, control various genes involved in regulating cholesterol homeostasis and lipid metabolism [4]. Acetyl-CoA carboxylase gene is a major downstream target of SREBP-1c and plays a key role in de novo lipogenesis (DNL) [5]. Also, peroxisome proliferator-activated receptors (PPARs) have pivotal roles in glucose and lipid hemostasis and intrahepatic lipid accumulation. PPAR-α is expressed dominantly in rat and human liver [6] Numerous genes are regulated by PPAR-α such as carnitine palmitoyltransferase1A (CPT1A) which is an important metabolic enzyme [7].

Currently, there are no approved therapeutic drugs available for use in patients with NAFLD/ NASH. Thus, there is an urgent need for developing novel medications. Medicinal plants have great potential for discovering new drugs for NASH treatment.

Capparis spinosa (Caper) belongs to the family of Capparidaceae and is a common medicinal plant used in many parts of the world to treat various diseases [8–10]. Administration of Caper fruit extract has been associated with inhibition of gluconeogenesis and fat accumulation in streptozotocin-induced diabetic rats, thus it might have positive effects on liver health and fatty liver [11]. However, its usefulness in NAFLD/ NASH and the possible underlying mechanisms have not yet been elucidated, and it warrants further investigations.

In this study, we evaluated the hepatoprotective effects of CS fruit extract in a rat model of HFD-induced NASH and possible mechanisms. Since fibrates are commonly used in the clinic to treat dyslipidemia, and several studies have shown their preventive efficacy in NAFLD progression to NASH [12], we compared the effects of CS with fenofibrate.

## Main text

### Methods

#### Providing CS aqueous extract

Capparis spinosa fruits were picked from wild plants growing in the Shooshtar region in Khuzestan province, southwest Iran. More details are in Additional file 1.

#### Providing high-fat emulsion

As mentioned by Yuhong Zou et al. high-fat emulsion (HF) was provided [13]. More details are in Additional file 1.

#### Providing chemicals

Fenofibrate was bought from the Sinadarou Company (Tehran, Iran). More details are in Additional file 1.

#### Animals and treatments

The male Wistar rats (n = 40) aged 6–7 weeks (180 ± 20 g) were prepared by the Experimental Animal Center, Ahvaz Jundishapur University of Medical Sciences. At first, animals were divided into two groups: the normal control group (NC group, n = 10) and the high-fat emulsion (HF group, n = 30). After 6 weeks, HF group was divided into three groups (n = 8), high-fat emulsion (HF group), high-fat emulsion plus fenofibrate 100 mg/kg body weight (HF + FENO group), or CS 20 mg/kg body weight (HF + CS group), respectively. More details are in Additional file 1.

#### Biochemical measurements

All detailed procedures of biochemical measurements are described in Additional file 1.

#### Gene expression analysis

The expression of SREBP1-c, acetyl*-*CoA carboxylase (ACC), PPARα, and carnitine palmitoyltransferase 1 (CPT1) mRNA was assessed by the real-time PCR technique. More detailed procedures are described in Additional file 1.

#### Histopathological evaluations

The sections of liver tissue were immediately obtained after the rats were sacrificed. The tissue specimens were fixed in 10% formalin solution. More details are in Additional file 1.

#### Statistical analysis

Quantitative data were noted as mean ± SD, and qualitative variables were noted as percentages. One-way analysis of variance (ANOVA) and Tukey's post hoc tests were used to compare quantitative variables. For the categorical data, the Chi-square test was done. The significance level for all tests was regarded at p ≤ 0.05. The analyses were handled by applying the GraphPad Prism software version 8.0.2.

## Results

### The impacts of treatments on liver index and histopathological parameters

**Fig. 1:**
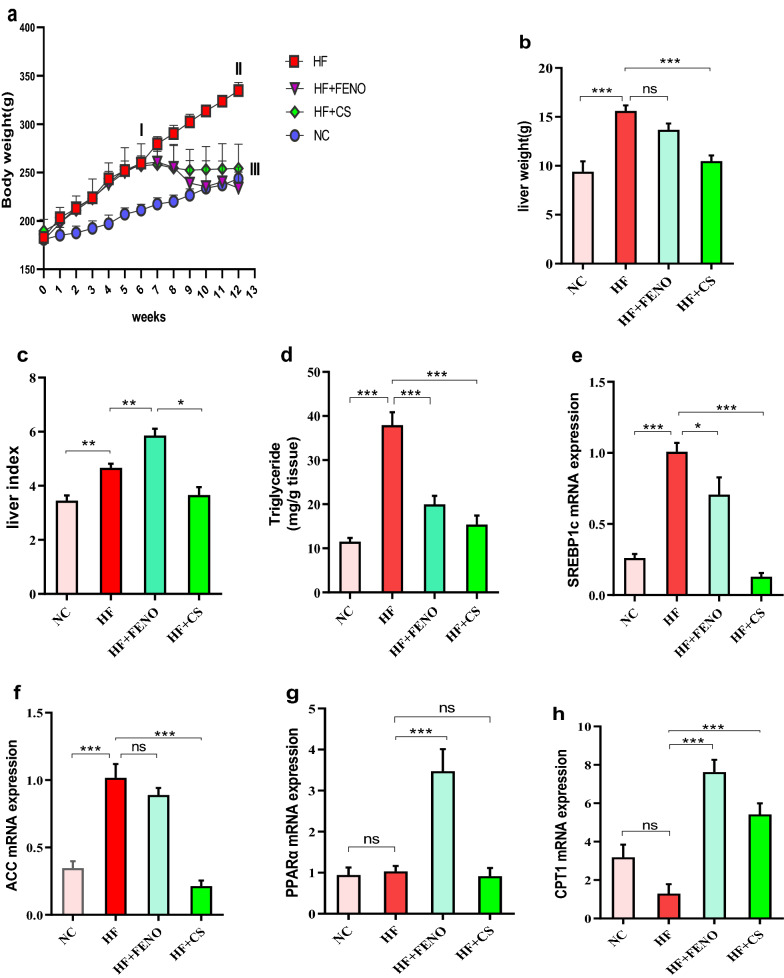
Effect of Capparis spinosa fruit extract and fenofibrate on the body weight (**a**), liver weight (**b**), liver index (**c**), liver TG content (**d**), sterol regulatory element-binding protein 1c (SREBP1c) (**e**), acetyl-CoA carboxylase (ACC) (**f**), peroxisome proliferator-activated receptor α (PPARα) (**g**), carnitine palmitoyltransferase 1 (CPT1) (**h**) mRNA expression in NASH model rats during 12 weeks of treatment. Bars represent the mean ± SD of the variables in each group (n = 8). NC, normal control; HF, high fat; CS, Capparis spinosa; FENO, fenofibrate. *p < 0.05; **p < 0.01; ***p < 0.001 and ns: nonsignificant. **I** Significantly different from NC at end of week 6; **II** Significantly different from NC after 12 weeks and **III** significantly different between NC, HF + FENO, and HF + CS vs. HF at end of week 12 (p < 0.001)

Based on Fig. 1a, after 12 weeks, HF group rats had a mean body weight of 334.71 ± 4.71 g in comparison with 243.57 ± 6.77 g in control rats, showing a considerable increase in body weight of HF group rats in comparison with the control group (p < 0.001). CS and fenofibrate treatment almost normalized this increase in body weight in HF + CS and HF + FENO groups. Moreover, liver weights in the HF group were significantly increased (p < 0.001) in comparison with the NC group. The treatment with CS for 42 consecutive days significantly decreased the liver weight in comparison with the HF group. Hence, there was no considerable difference between the fenofibrate treatment group, and the HF group found in the liver weight (Fig. 1b). Also, CS treatment considerably attenuated this liver index in comparison with the HF group (p < 0.05). More details are in Additional file 1.

### The impacts of treatments on the biochemical parameters

#### Liver performance tests

CS and fenofibrate treatments reversed the detrimental impacts of high-fat emulsion and normalized AST and ALT activity to levels in comparison with those in the NC group; thus, CS treatment showed more effectiveness in this regard in comparison with the fenofibrate (Table 1).

**Table 1:** Effect of daily administration of CS (20 mg/kg) and fenofibrate (100 mg/kg) on Serum biochemistry parameters of fatty liver rats

Parameters	NC	HF	HF + FENO	HF + CS	P-Value			
					HF VS. NC	HF + FENO VS. HF	HF + CS VS. HF	HF + CS VS. HF + FENO
AST (U/L)	36.21 ± 4.15	91.00 ± 18.06	73.41 ± 7.14	47.00 ± 5.11	< 0.001	0.0117	< 0. 001	0. 002
ALT (U/L)	28.75 ± 3.93	73.12 ± 6.43	51.68 ± 11.17	38.38 ± 5.72	< 0.001	0.0047	< 0. 001	0.0244
TG (mg/dl)	44.57 ± 8.14	115.10 ± 16.32	38.29 ± 09.66	48.25 ± 07.13	< 0.001	< 0. 001	< 0. 001	ns
TC (mg/dl)	71.57 ± 2.63	125.12 ± 7.59	83.37 ± 11.04	82.44 ± 7.90	< 0.001	< 0. 001	< 0. 001	ns
HDL-c (mg/dl)	44.00 ± 2.82	27.71 ± 5.03	32.86 ± 7.84	37.75 ± 4.68	< 0.001	ns	0.0071	ns
LDL-c (mg/dl)	25.66 ± 3.33	71.40 ± 7.25	48.06 ± 11.57	35.83 ± 6.51	< 0.001	< 0. 001	< 0. 001	0.0041
FFA (mg/dl)	9.99 ± 3.04	29.52 ± 4.37	36.63 ± 6.08	19.86 ± 4.01	< 0.001	< 0. 001	0.0014	< 0. 001
FBG (mg/dl)	105.70 ± 7.54	254.30 ± 8.95	160.1 ± 20.22	120.10 ± 8.72	< 0.001	< 0. 001	< 0. 001	< 0. 001
Insulin (mIU/L)	3.68 ± 1.07	3.079 ± 1.088	5.744 ± 0.79	3.327 ± 1.53	ns	0.0012	ns	0.0033
HOMA-IR	0.96 ± 0.26	1.925 ± 0.68	2.268 ± 0.41	1.04 ± 0.49	0.0059	ns	0.0130	0. 005

Values are expressed as the mean ± SD of parameters analyzed by one-way ANOVA and Tukey post hoc tests (n = 7 in each group)

*AST* aspartate aminotransferase, *ALT* alanine aminotransferase, *TG* triacylglycerol, *TC* total cholesterol, *HDL-c* high-density lipoprotein cholesterol, *LDL-c* low-density lipoprotein cholesterol, *FFA* free fatty acid, *FBS* fasting blood glucose, *HOMA-IR* homeostatic model assessment for insulin resistance, *NC* normal control, *HF* high fat, *CS* capparis spinosa, *FENO* fenofibrate

#### Serum lipid profile

Serum levels of TC, TG, LDL-C, and FFA in the HF group were significantly increased in comparison with the NC group, except for HDL-C. Treatment with CS and fenofibrate for six weeks in addition to the high-fat emulsion led to a diminution of these lipid parameters significantly (P < 0.001). In addition, the interventions improved the HDL-C levels (Table1).

#### Glycemic indices

Glycemic indices including fasting blood glucose and HOMA-IR decreased in response to CS but insulin was not affected by CS treatment. More details are in Additional file 1.

### Hepatic mRNA expression of SREBP-1c, ACC, PPARα, and CPT1

Regarding qPCR mRNA expression analysis in the post-treatment livers, we noticed the increased hepatic SREBP1c and ACC mRNA expression in the HF group in comparison with the NC group (Fig. 1**e**). Fenofibrate treatment significantly reduced the SREBP-1 expression in comparison with the HF group (p < 0.05). Hence, CS treatment could significantly and more effectively than fenofibrate reduce the elevated mRNA expression of SREBP-1c following 12 weeks of high-fat emulsion feeding (p < 0.001). Based on Fig. 1f, while a considerable reduction in the hepatic mRNA expression of ACC was found in the CS-treated rats (p < 0.001), it was not influenced considerably in the fenofibrate group in comparison with the HF group.

**Fig. 2:**
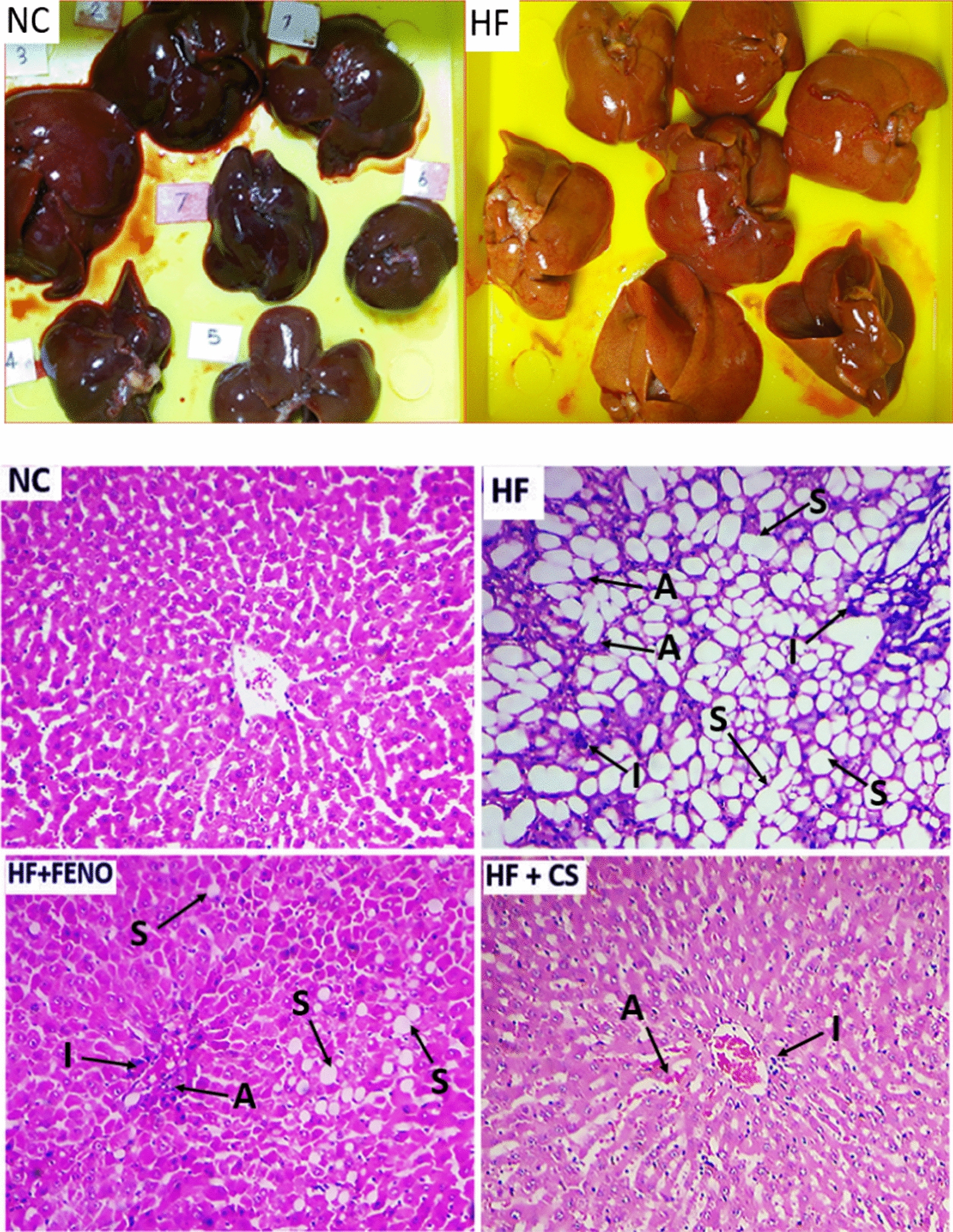
Upper panel: Macroscopic observation of the livers of the HF group showed a grossly larger and beige in color compared with the NC group. Lower panel: Representative images of hematoxylin–eosin-stained sections of liver tissue in different groups at the end of treatments with 100X magnification. A: accumulation of RBCs; I: inflammation; S: steatosis. *NC* normal control, *HF* high fat, *CS* Capparis spinose, *FENO* fenofibrate

The high-fat emulsion feeding did not change the mRNA expression of PPARα and CPT1 and no considerable difference between the NC group. 6 weeks of the intervention with PPARα agonist fenofibrate improved the hepatic mRNA levels of PPARα and its downstream target gene CPT1 considerably compared to the HF group (Fig. 1g). Thus, a considerable impact of CS on PPARα expression was not seen. Based on Fig. 1h, though the CPT1 expression answering to CS treatment was not as much as fenofibrate, it was considerably regarded in comparison with the HF group (p < 0.001) (Fig. 2).

## Discussion

In this study, we evaluated that CS improves non-alcoholic steatohepatitis through down-regulating SREBP-1c and a PPARα-independent pathway in high-fat diet-fed rats. Traditional herbal medicine has recently drawn much attention to the treatment of human diseases, including fatty liver [14–16]. We observed significant improvement in steatosis and triglyceride content of the liver tissue of the HF-fed rats treated with the CS (Fig. 2). A major predisposing factor in the pathogenesis of steatosis and triglyceride accumulation in the liver is insulin resistance which is associated with increased flux of fatty acid from adipose tissue to the liver [3]. In our study, the treatment of high fat-fed rats with CS decreased the HOMA-IR index which can describe the observed decrease in serum-free fatty acid in these animals. These findings indicate the protective and beneficial therapeutic effects of CS on liver damages associated with NASH. In comparison with CS, which improved glycemic parameters in our NASH model, fenofibrate did not represent beneficial effects on these parameters. This difference suggests that CS and fenofibrate may involve distinct mechanisms to improve NASH. However, in a previous study, we showed an important part of these mechanisms in which we observed that FGF21 increased in response to CS treatment thereby reducing steatosis, inflammation, and fibrosis in the NASH animal model [17]. To more elucidate the mechanism by which CS ameliorates steatosis, we analyzed the CS effects on the expression of the key regulatory genes of hepatic fat content. Among the involved genes, we investigated the changes in PPARα, CPT-1, SREBP-1c, and ACC expression. SREBP-1c is a crucial transcription factor involved in the regulation of lipid metabolism in the liver. There is credible evidence that a high-fat diet activates SREBP1c, and this activation plays an important role in the development and progression of NAFLD. Therefore, inhibition of SREBP1c is considered a therapeutic target for preventing and treatment of NAFLD [18, 19]. In the present study, high-fat diet feeding induced SREBP-1c while CS treatment inhibited its expression in our NASH model. These findings suggest the down-regulation of SREBP1c expression as an underlying mechanism by which CS can ameliorate NASH. To confirm the effect of CS on SREBP-1c function, we investigated the expression of acetyl-CoA carboxylase (ACC), as a downstream target of this transcription factor, in response to CS. Previous studies have shown that inhibition of ACC can significantly ameliorate fatty liver. Thus, pharmacologic inhibition of ACC is considered an attractive strategy for NAFLD treatment [20]. However, it has also been reported that ACC inhibition might result in hypertriglyceridemia through decreasing polyunsaturated fatty acids which further led to SREBP1 induction and decreased PPARα and CPT1 activity [21]. Our results demonstrated that CS treatment could down-regulate ACC expression in HF-fed rats with a fatty liver that was associated with a decrease in plasma TG.

PPARα is a dominant transcription factor in the liver, regulating numerous pathways involved in lipid metabolism including fatty acid activation, transport of fatty acid to the mitochondria, β-oxidation, and lipogenesis [22, 23]. There is growing evidence that indicates PPARα activation can hinder fatty liver development. Hence, pharmacologic PPARα agonists such as fenofibrate are proposed as therapeutic options for NAFLD [24, 25]. In our study, HF feeding did not change PPARα expression as compared to the NC rats. Following the previous studies [26, 27], fenofibrate treatment increased PPARα gene expression. However, we did not find a significant alteration in PPARα gene expression in response to CS treatment in HF-fed rats. This finding suggests that alternative mechanisms independent of PPARα expression might be involved in the observed CS effects.

For further investigation of the PPARα pathway in steatosis alleviation by the CS, we evaluated CPT1 expression as a target gene of PPARα. As a PPARα agonist, fenofibrate significantly increased CPT1 expression. Likewise, our results indicated that CS treatment could significantly up-regulate CPT1 expression in HF-fed rats. But, considering the non-significant effect of CS on PPARα expression, it seems that alternative pathways other than PPARα such as the AMPK pathway might be involved in the CS-induced CPT1 expression.

ALT and AST are considered the most sensitive and specific indicators of hepatocellular injury [3, 28, 29]. We found high levels of these enzymes in the HF group while the administration of CS reduced the elevated serum levels of ALT and AST. Following the decrease in liver enzymes, we found CS markedly diminished the fat deposition and lipid accumulation in liver tissues based on histopathological evaluations. Our results confirmed that CS administration can ameliorate body weight, liver weight, and liver index in high fat-fed rats.

## Conclusion

This study indicated that therapy with CS extract through the molecular mechanism, markedly improves hepatic lipid accumulation and steatohepatitis in NASH model rats and these beneficial effects were comparable to fenofibrate effects.

## Limitations

This study has not determined which of the active compounds of the CS is responsible for these effects, and it is necessary to examine in future studies.

## Supplementary Information

**Additional file 1:** Additional main text.

**Additional file 2:** ARRIVE guidelines.

**Additional file 3: Table S1. **Composition and caloric content of the high fat emulsion

**Additional file 4: Table S2.** Primer sequences of the target and reference genes amplification

## Data Availability

All data generated or analyzed during this study are included in this published article (and its Additional file 1).

## Abbreviations

NAFLD: Non-alcoholic fatty liver disease
NASH: Non-alcoholic steatohepatitis
CS: Capparis spinosa
HF: High fat
TG: Triglyceride
SREBP-1c: Sterol regulatory element-binding protein-1c
ACC: Acetyl-CoA carboxylase
PPARα: Peroxisome proliferator-activated receptor α
CPT1: Carnitine palmitoyltransferase I
DNL: De novo lipogenesis
STZ: Streptozotocin
HOMA-IR: Homeostatic model assessment of Insulin resistance
