# Supplementary material for: Capparis spinosa improves non-alcoholic steatohepatitis through down-regulating SREBP-1c and a PPARα-independent pathway in high-fat diet-fed rats
Source: BMC Res Notes. 2022 Oct 3;15:315. doi: 10.1186/s13104-022-06205-x (PMC9528135; doi:10.1186/s13104-022-06205-x)
Supplement: Supplementary file 1 — Additional file 1: Additional main text. [file 13104_2022_6205_MOESM1_ESM.docx]

**Additional file 1**

**Methods**

*Providing CS aqueous extract*

Determination and taxonomy assessment was done by Dr. Fereshteh Gol Fakhrabadi from Ahvaz Jundishapur University of Medical Sciences. Fruits were deposited at the Medicinal Plant Research Center, Ahvaz Jundishapur University of Medical Sciences (Ahvaz, Iran) with the voucher number A1805812FP.

Providing the aqueous extract was done in terms of the approach explained by Jalali et al. (1). In brief, CS fruits were washed with distilled water, dried at 40°C, and then powdered. To 10 g powdered fruits, 100 ml distilled water was added and stirred for 3 hours. The mentioned mixture was boiled for about 10 min and cooled for about 15 min. The aqueous extract was filtered by applying a 0.2 mm Millipore filter (Millipore 0.2 mm, St Quentin en Yvelines, France). The obtained filtrate was freeze-dried and stored at -20 °C for subsequent utilization. Fresh aqueous extracts were daily reconstituted immediately before the administration. The freeze-dried extract was reconstituted in 1.5 ml of distilled water and orally given to various groups with a 20 mg/kg dose, regarding Jalali et al. research (1).

*Providing high-fat emulsion*

The HF composition is noted in Table S1 which involved 77% fat, 14% total milk powder, and 9% carbohydrates. In the present formulation, the source of protein, carbohydrate, and fat are full milk powder, saccharose, and corn oil, respectively. Moreover, HF emulsion was supplemented with the vitamin and mineral mixture. The last emulsion was maintained at 4 ° C, and before utilization was heated and completely mixed in a 40 °C water bath.

*Providing Chemicals*

For oral performance, fenofibrate suspension was provided in 0.5% w/v sodium carboxymethylcellulose (Sigma, St. Louis, MO, USA) in distilled water. In 0.1M fresh cold citrate buffer solution at pH 4.2, the streptozotocin (Sigma, St. Louis, MO, USA) was obtained.

*Animals and treatments*

They were located in the plastic cages under the standard environmental situations at a room temperature of 24±1 °C with 55±5% humidity under 12-h light-dark cycles. Our research was done based on the ethical principal and national norms and standards to conduct medical research in Iran which was confirmed by the Ethics Committee of Research Center & Experimental Animal House, Ahvaz Jundishapur University of Medical Sciences.

At first, 40 rats were randomly (by a random number table) divided into two groups: the normal control group (NC group, n=10) and the HF group which achieved the high-fat emulsion (10 ml/kg, n=30) orally once per day and free achievement to a saccharose solution (18%). Passing 6 weeks, the rats of the HF group were injected intraperitoneally with a freshly provided solution of Streptozotocin (STZ) at a single low dose (30 mg/kg) to induce a more feature model of NASH. The gold standard for diagnosis of NAFLD and NASH is based on liver biopsy and histology that is characterized by hepatic steatosis, ballooning, and inflammation. Therefore, in the current study, inclusion criteria were the presence of histological features of steatosis or steatohepatitis, and exclusion criteria were any other liver disease. In the final part of the sixth week, two rats from the NC group and the HF group were sacrificed, and their liver was transformed in the lab for pathological investigation to ensure NASH development. After confirming the model, the pharmacological treatments were begun from the seventh experimental week which followed until the final of week 12 daily. To this objective, the HF group was divided by a random number table into three groups (n=8) to achieve either high-fat emulsion (HF group), high-fat emulsion plus fenofibrate 100mg/kg body weight (HF+FENO group), or CS 20 mg/kg body weight (HF+CS group). Fenofibrate and CS were suspended in 0.5 % carboxymethylcellulose (CMC) solution and were daily given at 10:00 a.m. in a volume equal to 1.5 ml/kg via gavage. The rats in the HF group received equal volumes of 0.5 % CMC solution (drug vehicle) while the NC rats were given the same volume of distilled water via gavage similarly. All rats achieved a standard rodent chow diet and water through the study course. At the final treatments, regarding 14 h fasting, all rats were anesthetized through intraperitoneal injection of ketamine hydrochloride (90 mg/kg) and xylazine (10 mg/kg) according to their body weight. they were kept in deep anesthesia using the nose cone method. The blood samples were gathered via the cardiac puncture. The livers were immediately removed, weighed, and washed with normal saline and were flash-frozen in liquid nitrogen which was kept at -190 ºC for further analysis of gene expression. The liver index was computed from the ratio of liver weight/body weight.

*Biochemical measurements*

Serum alanine aminotransferase (ALT), aspartate aminotransferase (AST), triglyceride (TG), total cholesterol (TC), high-density lipoprotein (HDL-C), low-density lipoprotein (LDL-C), and glucose in serum were done by the Roche 6000 auto-analyzer applying the corresponding assay kits. The levels of free fatty acids (FFA) and insulin in serum were identified by applying a commercial analysis kit and an enzyme-linked immunoassay kit (DRG Diagnostics, Marburg, Germany), respectively, following instructions of the manufacturer. Furthermore, the hepatic TG content was enzymatically measured by a colorimetric-specific kit (Cayman Chemical, USA).

*Gene expression analysis*

In brief, the total RNA from the frozen livers was separated with the FastPure™ RNA Kit from Takara Bio (Otsu, Japan) and cDNA synthesis was done applying the PrimeScript RT reagent Kit (Takara Bio, Otsu, Japan) following the procedures obtained by the manufacturer. Real-time PCR was done with an SYBR Green PCR kit (Takara Bio, Otsu, Japan) on QuantStudio™ 3 Real-Time PCR System (ABI Applied Biosystems) based on the manufacturer’s protocol. Comparative quantification of gene expression was utilized as noted in the manual applying β-actin as an internal control. The primer sequences were indicated in Table S 2.

*Histopathological evaluations*

Sections of the liver were dehydrated and embedded with paraffin which was stained with hematoxylin-eosin (HE) for histopathological assessments to survey the hepatic steatosis, inflammation, necrosis, and fibrosis. Steatosis was graded due to hepatocytes percentage including the macrovesicular fat (Grade 1: 0–25%; grade 2: 26–50%; grade 3: 51–75%; grade 4, 76–100%) (2). The degree of inflammation, necrosis, and fibrosis was explained by the mean of 10 various fields in every slide which were grouped on a scale of 0–3 (0: normal; 1: mild; 2: moderate; 3: severe) which was described by Avni et al. (3).

**Results**

*The impacts of treatments on liver index and histopathological parameters*

Moreover, the liver index was increased answering to the fenofibrate treatment (p< 0.01) in comparison with high-fat emulsion alone (Fig 1.c).

The liver triglyceride content was markedly increased in the HF group in comparison with the NC group. CS treatment for six weeks was efficient to inhibit hepatic triglyceride accumulation. Furthermore, the intervention with fenofibrate normalized hepatic TG content in comparison with the HF group (Fig 1.d).

HF-fed rats enhanced the severe degrees of steatosis. The livers of these rats were grossly larger than those in the NC group which were beige (Fig 2, upper panel). The severe steatosis was approved by hematoxylin-eosin staining of liver sections. Passing 6 weeks of CS and fenofibrate treatment, considerable enhancement was seen in the steatosis and aspects of necro-inflammation of hepatic tissue (Fig.2, lower panel).

*Glycemic indices*

High-fat emulsion administration together with a low dose STZ injection resulted in a 2.4-fold increase in the fasting blood glucose (FBG) level in the HF group in comparison with the control group. Plasma insulin levels in the HF group stayed without change which gave rise to a 2-fold increase in the HOMA index in comparison with the control group (Table1). The treatment with CS and fenofibrate significantly (P <0.001) restored the increase in FBG levels. The insulin level did not alter answering to CS vs. HF emulsion alone which led to decreasing HOMA-IR index in the CS group in comparison with the HF group (p <0.05). While, fenofibrate treatment increased the insulin level which consequently reduced the HOMA-IR index in comparison with the HF group (Table 1).

1. Jalali MT, Mohammadtaghvaei N, Larky DA. Investigating the effects of Capparis spinosa on hepatic gluconeogenesis and lipid content in streptozotocin-induced diabetic rats. Biomedicine & pharmacotherapy. 2016;84:1243-8. <https://doi.org/10.1016/j.biopha.2016.10.061>

2. Kirsch R, Clarkson V, Shephard EG, Marais DA, Jaffer MA, Woodburne VE, et al. Rodent nutritional model of non‐alcoholic steatohepatitis: species, strain and sex difference studies. Journal of gastroenterology and hepatology. 2003;18(11):1272-82. <https://doi.org/10.1046/j.1440-1746.2003.03198.x>

3. Avni Y, Shirin H, Aeed H, Shahmurov M, Birkenfeld S, Bruck R. Thioacetamide-induced hepatic damage in a rat nutritional model of steatohepatitis. Hepatology research. 2004;30(3):141-7. <https://doi.org/10.1016/j.hepres.2004.08.004>
