# Supplementary material for: Capparis spinosa improves non-alcoholic steatohepatitis through down-regulating SREBP-1c and a PPARα-independent pathway in high-fat diet-fed rats
Source: BMC Res Notes. 2022 Oct 3;15:315. doi: 10.1186/s13104-022-06205-x (PMC9528135; doi:10.1186/s13104-022-06205-x)
Supplement: Supplementary file 3 — Additional file 3: Table S1. Composition and caloric content of the high fat emulsion [file 13104_2022_6205_MOESM3_ESM.docx]

Table S1.Composition and caloric content of the high fat emulsion

| Corn oil (g) | 400 |
| --- | --- |
| Saccharose (g) | 150 |
| Total milk powder (g) | 80 |
| Cholesterol (g) | 100 |
| Sodium deoxycholate (g) | 10 |
| Tween 80 (g) | 36.4 |
| Propylene glycol (g) | 31.1 |
| Vitamin mixture (g) | 2.5 |
| Cooking salt (g) | 10 |
| Mineral mixture (g) | 1.5 |
| Distilled water (ml) | 300 |
| **Total energy (kcal/l)** | **4342** |
