# Supplementary material for: Capparis spinosa improves non-alcoholic steatohepatitis through down-regulating SREBP-1c and a PPARα-independent pathway in high-fat diet-fed rats
Source: BMC Res Notes. 2022 Oct 3;15:315. doi: 10.1186/s13104-022-06205-x (PMC9528135; doi:10.1186/s13104-022-06205-x)
Supplement: Supplementary file 4 — Additional file 4: Table S2. Primer sequences of the target and reference genes amplification [file 13104_2022_6205_MOESM4_ESM.docx]

Table S 2. Primer sequences of the target and reference genes amplification

| **Gene** | **Forward primer** | **Reverse primer** |
| --- | --- | --- |
| **PPAR**α | 5'- CCCCACTTGAAGCAGATGACC -3' | 5'- CCCTAAGTACTGGTAGTCCGC -3' |
| **CPT-1** | 5'-GCTCGCACATTACAAGGACAT-3′ | 5'-TGGACACCACATAGAGGCAG-3′ |
| **SREBP1-c** | 5′- GGAGCCATGGATTGCACATT -3 | 5′- GCTTCCAGAGAGGAGCCCAG -3 |
| **ACC** | 5'-CGCTGCGGTCAAGTGT-3′ | 5′-CGTTGGCGTAGTTGTTATT-3′ |
| **β-Actin** | 5′-CCCATCTATGAGGGTTACGC-3 | 5′-TTTAATGTCACGCACGATTTC-3 |
